# Functional Plasticity of Gamma Delta T Cells and Breast Tumor Targets in Hypoxia

**DOI:** 10.3389/fimmu.2018.01367

**Published:** 2018-06-15

**Authors:** Gabrielle M. Siegers, Indrani Dutta, Raymond Lai, Lynne-Marie Postovit

**Affiliations:** ^1^Department of Oncology, University of Alberta, Edmonton, AB, Canada; ^2^Department of Anatomy and Cell Biology, Robarts Research Institute, Western University, London, ON, Canada

**Keywords:** gamma delta T cells, plasticity, hypoxia, breast cancer, tumor evasion, MHC class I polypeptide-related sequence A

## Abstract

Interactions between immune and tumor cells in the tumor microenvironment (TME) often impact patient outcome, yet remain poorly understood. In addition, the effects of biophysical features such as hypoxia [low oxygen (O_2_)] on cells within the TME may lead to tumor evasion. Gamma delta T cells (γδTcs) naturally kill transformed cells and are therefore under development as immunotherapy for various cancers. Clinical trials have proven the safety of γδTc immunotherapy and increased circulating γδTc levels correlate with improved patient outcome. Yet, the function of γδTc tumor infiltrating lymphocytes in human breast cancer remains controversial. Breast tumors can be highly hypoxic, thus therapy must be effective under low O_2_ conditions. We have found increased infiltration of γδTc in areas of hypoxia in a small cohort of breast tumors; considering their inherent plasticity, it is important to understand how hypoxia influences γδTc function. *In vitro*, the cell density of expanded primary healthy donor blood-derived human γδTc decreased in response to hypoxia (2% O_2_) compared to normoxia (20% O_2_). However, the secretion of macrophage inflammatory protein 1α (MIP1α)/MIP1β, regulated on activation, normal T cell expressed and secreted (RANTES), and CD40L by γδTc were increased after 40 h in hypoxia compared to normoxia concomitant with the stabilization of hypoxia inducible factor 1-alpha protein. Mechanistically, we determined that natural killer group 2, member D (NKG2D) on γδTc and the NKG2D ligand MHC class I polypeptide-related sequence A (MICA)/B on MCF-7 and T47D breast cancer cell lines are important for γδTc cytotoxicity, but that MIP1α, RANTES, and CD40L do not play a direct role in cytotoxicity. Hypoxia appeared to enhance the cytotoxicity of γδTc such that exposure for 48 h increased cytotoxicity of γδTc against breast cancer cells that were maintained in normoxia; conversely, breast cancer lines incubated in hypoxia for 48 h prior to the assay were largely resistant to γδTc cytotoxicity. MICA/B surface expression on both MCF-7 and T47D remained unchanged upon exposure to hypoxia; however, ELISAs revealed increased MICA shedding by MCF-7 under hypoxia, potentially explaining resistance to γδTc cytotoxicity. Despite enhanced γδTc cytotoxicity upon pre-incubation in hypoxia, these cells were unable to overcome hypoxia-induced resistance of MCF-7. Thus, such resistance mechanisms employed by breast cancer targets must be overcome to develop more effective γδTc immunotherapies.

## Introduction

Low oxygen (O_2_) levels (hypoxia) characterize the microenvironment of many solid tumors, occurring as a consequence of structurally disorganized blood vessels and tumor growth that exceeds the rate of vascularization. Hypoxia is common within breast cancers, which have a median O_2_ concentration of 1.4%, as compared to ~9.3% for normal breast tissue (1). In response to hypoxia, cells express genes that are essential for their survival. In tumor cells, this O_2_-regulated gene expression leads to more aggressive phenotypes, including those that increase the ability of cells to resist therapy, recruit a vasculature and metastasize (2–4). Accordingly, there is a growing body of evidence correlating tumor hypoxia with poor clinical outcome for patients with a variety of cancers (5–7). O_2_ availability has also been shown to regulate immune editing, allowing cancer cells to evade the immune system *via* a variety of mechanisms (8). For example, hypoxia upregulates hypoxia inducible factor 1-alpha (HIF1α)-dependent ADAM10 expression resulting in MHC class I polypeptide-related sequence A (MICA) shedding from the surface and decreased lysis of tumor cells (9). While many studies have focused on myeloid-derived suppressor cells or conventional CD8+ T cells (8), so far none have considered the impact of tumor hypoxia on gamma delta T cells (γδTcs).

While γδTc kill cancer cell lines, derived from both hematological and solid tumors alike [reviewed in Ref. (10)], it is unclear whether they are still active cancer killers when confronted with the harsh and immunosuppressive tumor microenvironment (TME) (10–13). We have focused on breast cancer, since there have been conflicting reports in the literature with respect to γδTc function in this disease. While *in vitro* studies clearly show that γδTc are able to kill breast cancer cell lines MDA-MB231, MCF-7, and T47D (14–16), it is unclear as to whether γδTc retain their cytotoxic properties once exposed to the breast tumor TME (11).

Here, we set out to determine how γδTc behave under low O_2_, a TME factor likely encountered by γδTc in many malignancies. Carbonic anhydrase IX (CAIX) is a transmembrane protein that catalyzes the reversible hydration of carbon dioxide. It is expressed in response to hypoxia and is thus used as a surrogate marker for hypoxia (17). High CAIX expression indicates poor prognosis in many cancers, including breast cancer (18–20). Breast cancer cell lines express MICA, a ligand for the natural killer group 2, member D (NKG2D) receptor expressed by γδTc and implicated in γδTc cytotoxicity (21–25). Thus, we have further explored the integral role for NKG2D/MICA in γδTc cytotoxicity against breast cancer cell lines under hypoxia and normoxia.

Since γδTc are being developed for cancer immunotherapy (26–31), and have shown both safety and even some efficacy—despite advanced disease stage—in a Phase I trial for breast cancer (32), it is imperative that we learn how the TME impacts the function of γδTc infiltrating breast and other solid tumors.

## Materials and Methods

### Ethics Statement

This study was carried out in accordance with the recommendations of the Research Ethics Guidelines, Health Research Ethics Board of Alberta—Cancer Committee with written informed consent from all subjects. All subjects gave written informed consent in accordance with the Declaration of Helsinki. The protocol was approved by the Health Research Ethics Board of Alberta—Cancer Committee.

### Patients and Tissues

We assessed 17 surgically resected breast tumors from cancer patients diagnosed at the Cross Cancer Institute, Edmonton, AB, Canada from 1997 to 1998. Patient and tumor characteristics are listed in Table 1.

**Table 1:** Characteristics of breast cancer cohort.

*n* = 17	*n* (%)	Median (range)
Age at diagnosis		51 (40–69)
Histology		
Invasive ductal carcinoma	14 (82)	
Invasive non-ductal tubular	1 (6)	
Invasive non-ductal mucinous	1 (6)	
Non-invasive	1 (6)	
Tumor size (cm)		
<2	11 (65)	1.4 (0.2–5.5)
2–5	4 (24)	
>5	1 (6)	
Not specified	1 (6)	
Tumor grade		
1/3	4 (24)	
2/3	5 (29)	
3/3	8 (47)	
Nodal status		
Positive	9 (53)	
Negative	8 (47)	
Estrogen receptor		
Positive	12 (71)	
Negative	3 (18)	
Not available	2 (12)	
Progesterone receptor		
Positive	10 (59)	
Negative	5 (29)	
Not available	2 (12)	

### Immunohistochemistry

Anti-human T cell antigen receptor (TCR)δ staining was performed as reported (33). Briefly, 4.5 µm serial sections from formalin-fixed paraffin-embedded tumors were melted on a slide warmer at 60°C for a minimum of 10 min followed by de-paraffinization using a fresh Xylenes (Thermo Fisher Scientific, Burlington, ON, Canada) bath. Sections were then hydrated with a series of graded ethanol (100, 95, 70, and 60%) followed by brief incubation in water, then tris-buffered saline plus 0.05% Tween-20 (TBST). Antigen retrieval was performed at 100°C for 20 min in target retrieval solution pH 9 (DAKO North America, Carpinteria, CA, USA). After cooling to room temperature, tissues were circled with an ImmEdge pen (Vector Laboratories, Burlingame, CA, USA) and blocked with Peroxidase Block (DAKO) for 5 min. Slides were washed in TBST for 5 min then blocked with Protein Block Serum Free (DAKO) for 10 min. Protein block was gently removed and replaced with 1:150 dilution of mouse monoclonal anti-human TCRδ antibody (clone H-41, Santa Cruz Biotechnology, Dallas, TX, USA) or 1:50 dilution of rabbit monoclonal anti-human CAIX [clone EPR4151(2), abcam, Cambridge, MA, USA] or corresponding isotype control diluted to the same antibody concentration; all dilutions were made in antibody diluent (DAKO). Known positive controls and isotype controls were included with each batch to ensure quality control of staining. Sections were incubated in a humidified chamber for 30 min at 25°C. Slides were then rinsed and washed five times in TBST for 5 min. Slides were then incubated with 100 µl secondary antibody, labeled polymer—horseradish peroxidase (HRP) anti-mouse or—HRP anti-rabbit (DAKO), for 60 min at room temperature in the humidified chamber. Washing was done as before, and then slides were treated with 75–100 µl 3,3′-diaminobenzidine chromogen solution (DAKO) for 8–10 min before the reaction was stopped by rinsing in water. Hematoxylin (DAKO) counterstaining was performed, slides were rinsed in water and then dehydrated using a series of graded ethanol (60, 70, 95, and 100%). Slides were then cleared with Xylenes, dried and coverslips mounted with VectaMount permanent mounting medium (Vector Laboratories).

### Assessment of CAIX Expression and γδTc Infiltration

Light microscopy and semi-quantitative scoring for CAIX was performed by a single pathologist; scores were 0, no staining; 1, weak and/or very focal staining; 2+, strong but focal staining; and 3, strong and extensive staining. Serial sections stained for TCRγδ and CAIX were scanned. Areas of CAIX-positivity and negativity were defined, and images from slides superimposed to enable counting of γδTc in CAIX-positive and -negative areas. Five consecutive areas within each region were quantified for the frequency of γδTc infiltration.

### Primary γδTc

We established and maintained primary human γδTc cultures as described (34). Briefly, healthy donor blood was diluted with phosphate buffered saline (PBS) and peripheral blood mononuclear cells (PBMCs) isolated using density gradient separation (Lymphoprep, Stem Cell Technologies, Vancouver, BC, Canada). PBMCs were cultured in a humidified atmosphere at 37°C with 5% CO_2_ at 1 × 10^6^ cells/ml in RPMI complete medium containing 1 µg/ml Concanavalin A (Sigma-Aldrich, Oakville, ON, Canada), 10% fetal bovine serum (FBS), 1× MEM NEAA, 10 mM HEPES, 1 mM Sodium Pyruvate (all Invitrogen, Burlington, ON, Canada), and 10 ng/ml recombinant human interleukin (IL)-2 and IL-4 (Miltenyi Biotec, Auburn, CA, USA). Cells were counted and viability assessed *via* Trypan Blue Exclusion Assay (Invitrogen/Thermo Fisher Scientific, Waltham, MA, USA); fresh medium and cytokines added to adjust density to 1 × 10^6^ cells/ml every 3–4 days. After 1 week, αβ T cells were labeled with anti-TCRαβ PE antibodies (BioLegend, San Diego, CA, USA) and anti-PE microbeads (Miltenyi Biotec), and depleted after filtering (50 µm Cell Trics filter, Partec, Görlitz, Germany) and passing over an LD depletion column (Miltenyi Biotec). γδTcs, which did not bind to the column, were further cultured in complete medium plus cytokines (as above). For cytotoxicity and blocking experiments, γδTc cultures were used on days 19–21, as they were most cytotoxic then. Some hypoxia experiments were done at earlier time points. Donor cultures are identified as follows: donor number culture letter-culture day; thus, 7B-13 = the second culture derived from donor 7 on day 13. Culture purities and subset compositions are shown in Table S1 in Supplementary Material.

### Breast Cancer Cell Lines

Human breast carcinoma cell lines, MCF-7 and T47D, were obtained from the American Type Culture Collection (ATCC, Manassas, VA, USA) and maintained as per ATCC guidelines. For surface marker staining of breast cancer cell lines, cells were harvested by washing with PBS followed by dissociation in Accutase (Sigma-Aldrich) for 20 min at 37°C.

### Hypoxia Experiments

To examine the effects of hypoxia, cells were cultured in O_2_ concentrations as indicated for 40–48 h using an X3 Xvivo Closed Incubation System (BioSpherix). After incubation under normoxic or hypoxic conditions, cell culture supernatants were collected, chilled on ice, and then frozen at −80°C until further analysis; harvested cells were used in cytotoxicity assays or stained for flow cytometric analysis. In some cases, cells were cold harvested, pellets frozen on dry ice, and stored at −80°C until lysis for Western blotting.

### Flow Cytometry

#### Antibodies

For surface marker staining of γδTc, the following anti-human antibodies from BioLegend, unless otherwise indicated, were employed: TCRγδ PE (clone B1, 1:25); TCR Vδ1 FITC (Miltenyi, clone REA173, 1:10); TCR Vδ2 PerCP (clone B6, 1:25); NKG2D APC (BD Biosciences, Mississauga, ON, Canada, 1:25); CD56 FITC (clone MEM-188, 1:5); CD69 AF700 (clone FN50, 1:4); CD94 FITC (clone DX22, 1:5); CD95 APC (clone DX2, 1:100); HLA ABC PE (clone W6/32, 1:10); FasL PE (clone NOK-1, 1:5); and CD40L APC (clone 24–31, 1:5).

Anti-human MICA/B PE (BioLegend, clone 6D4, 0.1 µg) was used to stain breast cancer cell lines.

#### Surface Marker Staining

Gamma delta T cell and breast cancer cell lines were adjusted to 10 × 10^6^ cells/1 ml, stained with 1 μl/10^6^ cells Zombie Aqua fixable viability dye in PBS (ZA, BioLegend) for 15–30 min at room temperature in the dark. γδTc were stained directly with fluorochrome-conjugated antibodies diluted in FACS buffer [PBS containing 1% FBS and 2 mM EDTA (Invitrogen)] as indicated above. Breast cancer cell lines at 10 × 10^6^ cells/ml were blocked in FACS buffer containing 50 µl/ml Trustain FcX (BioLegend) and incubated on ice for 30 min prior to antibody incubation. After blocking, cells were centrifuged and supernatants removed, leaving 10 µl FACS buffer plus block/10^6^ cells. Antibodies and FACS buffer were added to 20 µl, and cells incubated on ice 15–20 min followed by washing. All cells were fixed in FACS buffer containing 2% paraformaldehyde (Sigma-Aldrich), stored at 4°C and acquired within 1 week.

#### Flow Cytometer Specifications

Cells were analyzed using a FACS CANTO II (Becton Dickinson, Mississauga, ON, Canada) equipped with an air-cooled 405-nm solid state diode, 30 mW fiber power output violet laser, with 450/50 and 510/50 band pass (BP) [502 long pass (LP) detector]; a 488-nm solid state, 20-mW blue laser with 530/30 BP (502 LP), 585/42 BP (556 LP), 670 LP (655 LP), and 780/60 BP (735 LP) filters; and a 633-nm HeNe, 17-mW red laser with 660/20 BP and 780/60 BO (735 LP) filters. Calibration was performed with CS&T beads (Becton Dickenson, Mississauga, ON, Canada). Live singlets were gated based on forward and side-scatter properties. Fluorescence minus one (FMO) controls were used to set gates. Analysis was performed using FlowJo^©^ software (Tree Star, Ashland, OR, USA, Version 10.0.8r1).

#### Cytokine Arrays

The Proteome Profiler Human Cytokine Array Kit, Panel A (R&D Systems, Minneapolis, MN, USA) was used to detect proteins secreted by γδTc cultured under normoxic or hypoxic conditions. Undiluted culture supernatants were used in these assays, which were carried out according to the manufacturer’s instructions. Analysis of resulting films was done as follows: pixel intensities were measured using FIJI software (ImageJ Version 2.0.0-rc-15/1.49m) using a consistent circular region of interest; measured values from duplicate spots were subtracted from 255. The average intensity from the two negative spots was subtracted from all values to obtain net values. The intensities of the six reference spots (positive controls) were averaged and a multiplier was defined for each array (normalized to the array with the lowest pixel intensity). Values were adjusted accordingly and then values for the duplicates were averaged. Finally, ratios were calculated for each cytokine, normalized to normoxia.

#### ELISAs

1–2 ml aliquots of culture supernatants stored at −80°C were thawed on ice. Halt™ Protease and Phosphatase Inhibitor Cocktail (PIC, Thermo Fisher Scientific) was added to samples prior to use in ELISAs or further storage at 4°C. The following ELISA kits were used: ELISA MAX Deluxe regulated on activation, normal T cell expressed and secreted (RANTES/CCL5) (BioLegend), Human macrophage inflammatory protein 1α (MIP1α) and Human CD40L Quantikine ELISA kits (R&D Systems), and Human MICA ELISA Kit (abcam). For RANTES and CD40L ELISAs, culture supernatant samples were diluted up to 16-fold to obtain readings within range (1:2, 1:4, 1:8, 1:16). For MIP1α ELISAs, samples were diluted up to 1:20. For MICA ELISAs, culture supernatants stored at −80°C were thawed overnight in at 4°C, then 4 ml applied to Amicon Ultra-4 10 K spin columns (Merck-Millipore, Carrigtwohill, Ireland) that were subsequently centrifuged at 3,000 *g* for 2 h at 12°C. Concentrated media was then transferred into 1.5 ml Eppendorf tubes and diluted to 200 and 20 µl of a 1:10 dilution of PIC were added. For the ELISA, 100 µl per well were assayed in duplicate. All ELISAs were done according to the manufacturer’s instructions. Absorbance at 450 and 550 nm was measured using a FLUOstar Omega plate reader (BMG Labtech, Offenburg, Germany) with Omega Software version 5.11. The difference linear regression fit of the standard curve was used for concentration calculations. ELISA data were normalized to γδTc cell numbers and culture volumes.

#### Immunoblotting

Cell lysates were prepared by mixing γδTc with M-PER Mammalian Protein Extraction Reagent (Thermo Fisher Scientific) containing PIC at 10 µl lysis buffer per million γδTc followed by incubation at room temperature for 10 min. Lysates were then centrifuged at 13,000 rpm for 15 min 4°C, after which supernatants were transferred to fresh tubes and 5× reducing sample buffer [0.0625 M Tris/HCl pH6.8, 2% SDS, 20% glycerol, 0.05% β-mercaptoethanol, 0.025% (w/v) Bromophenol Blue] added. Samples were boiled 5 min, cooled, and briefly centrifuged in a benchtop centrifuge prior to running on 10 or 12% SDS-PAGE gels. Proteins were transferred onto Immobilon-FL PVDF membranes (Millipore) using the Trans-Blot Turbo Transfer System (Bio-Rad, Mississauga, ON, Canada). The high molecular weight (MW) program was used when transferring proteins for HIF1α detection. Otherwise, the mixed MW program was used. Membranes were blocked 40 min in 3% milk in TBST, followed by overnight incubation in primary antibody baths at 4°C. After washing, membranes were incubated with the corresponding species-specific HRP-labeled secondary antibody for 1 h, followed by further washing and then detection using Clarity™ Western ECL Substrate (Bio-Rad). Primary antibodies were diluted in PBS containing 2% bovine serum albumin and 0.05% sodium azide at the following dilutions: 1:500 mouse anti-human HIF-1α (clone MOP1, BD Biosciences); 1:2,000 goat anti-human CCL3/MIP1α (R&D Systems); 1:1,000 mouse anti-human/primate CCL5/RANTES (Clone #21418, R&D Systems); 1:500 mouse anti-human CD40 ligand/TNFSF5 (Clone #40804, R&D Systems); 1:2,000 rabbit anti-human β-Actin (Cell Signaling Technologies, Danvers, MA, USA). Secondary antibodies were diluted in blocking buffer as follows: 1:10,000 goat anti-mouse IgG HRP (Bio-Rad); 1:20,000 goat anti-rabbit IgG HRP (Bio-Rad); and 1:1,000 donkey anti-goat IgG HRP (R&D Systems).

#### Quantification of Bands on Western Blots

Band intensities for CD40L, MIP1α, and RANTES were measured using FIJI software (ImageJ Version 2.0.0-rc-15/1.49m) on converted grayscale images using consistent rectangular regions of interest. Measured values for bands and background (region of same size beneath each band) were subtracted from 255, then background was subtracted from bands to obtain net values for protein bands of interest and loading control bands (actin). The ratios of protein bands to loading control bands were then calculated. In the case of CD40L and RANTES, these values were multiplied by 10 to obtain values between 0.1 and 10. For calculation of induction, hypoxia values were divided by normoxia values, and average values for each protein were plotted. Calculations were done in Microsoft Excel version 15.3 (Microsoft, Redmond, WA, USA).

### Cytotoxicity Assays

#### Target Cell Labeling With Calcein AM (CalAM)

As per the manufacturer’s instructions, target cells were labeled with 5 µM CalAM (Invitrogen/Thermo Fisher Scientific). Cells were diluted to 30,000 cells/100 μl medium for cytotoxicity assays.

#### Blocking Antibodies

The following anti-human antibodies were used: LEAF purified anti-NKG2D (BioLegend, Clone 1D11); anti-human CCL3/MIP1α (R&D Systems); anti-human/primate CCL5/RANTES (Clone #21418, R&D Systems); and anti-human CD40 ligand/TNFSF5 (Clone #40804, R&D Systems). Mouse IgG (Sigma-Aldrich) was used as a control.

#### Blocking/Cytotoxicity Assay

For blocking and cytotoxicity assays, 6 × 10^6^ cells/ml γδTc cells were re-suspended in complete medium: RPMI 1640 plus 10% heat-inactivated FBS; 10 mM HEPES; 1× MEM NEAA; 1 mM sodium pyruvate; 50 U/ml penicillin–streptomycin; and 2 mM l-glutamine, all purchased from Invitrogen. Blocking antibodies were added at 6 µg mAb per 600 µl cell suspension/test in Eppendorf tubes, then plated at 100 μl/well in a 96-well round-bottomed plate and incubated at 37°C for 30 min. Thereafter, 100 µl CalAM-labeled targets were added. For cytotoxicity assays, the effector:target (E:T) ratio is indicated; blocking assays were done at 20:1. Co-cultures were incubated at 37°C for 4 h, after which plates were centrifuged and supernatants transferred to black clear-bottom 96-well (flat) plates (Costar, VWR International, Edmonton, AB, Canada). CalAM fluorescence was then detected on a FLUOstar Omega, BMG labtech fluorimeter. Controls were untreated and IgG-treated cells (for blocking assays), CalAM-labeled target cells incubated alone (spontaneous release) as well as 0.05% Triton-X 100 (Thermo Fisher Scientific)-treated cells (maximum release). The calculation for percent lysis is: [(test-spontaneous release)/(maximum-spontaneous release)] × 100%.

#### Statistics

**Figure 1:**
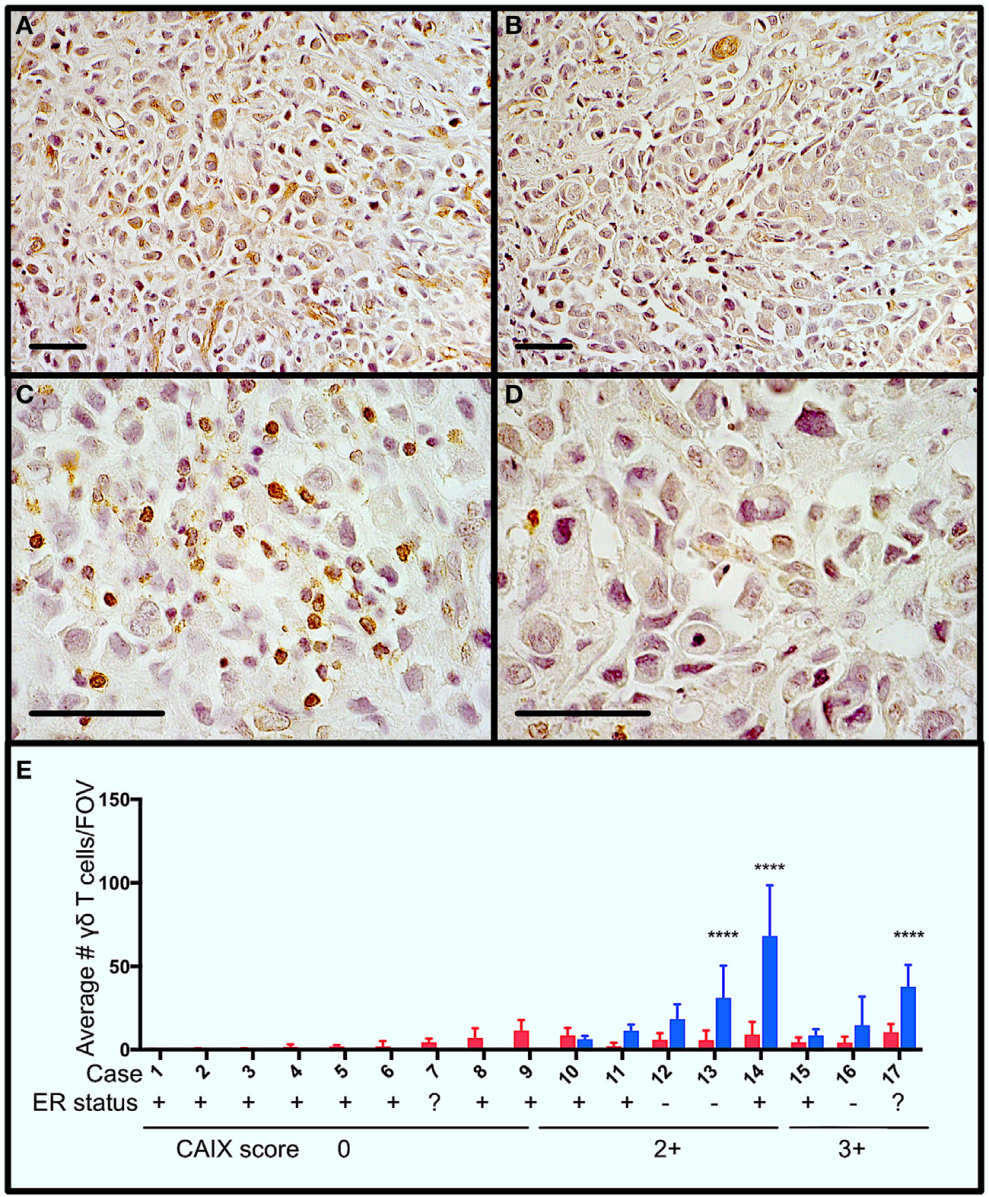
Gamma delta T cells (γδTcs) are present in areas of hypoxia in estrogen receptor positive (ER+) breast tumors. Serial sections from ER+ breast tumors were stained for carbonic anhydrase IX (CAIX) and T cell antigen receptor δ. **(A)** Example of CAIX-positive staining at 400× magnification from case 14; **(B)** CAIX-negative field of view (FOV) from the same slide as in **(A)**; **(C)** γδTc in the same area as **(A)** at 1,000× magnification; and **(D)** γδTc in the same area as **(B)** at 1,000× magnification. Scale bars are 50 µm. Brown indicates positive staining. **(E)** Parallel staining for γδTc and CAIX suggests that γδTc infiltration increases in hypoxic regions. CAIX scoring is indicated below the case numbers: 0 = no staining; 1 = weak and/or very focal staining; 2+ = strong but focal staining; and 3 = strong and extensive staining. Quantification and statistical analysis of γδTc frequency in CAIX-positive versus -negative regions (blue and red bars, respectively) reveal significantly increased γδTc infiltration in hypoxic regions (two-way ANOVA, ****P < 0.0001).

**Figure 2:**
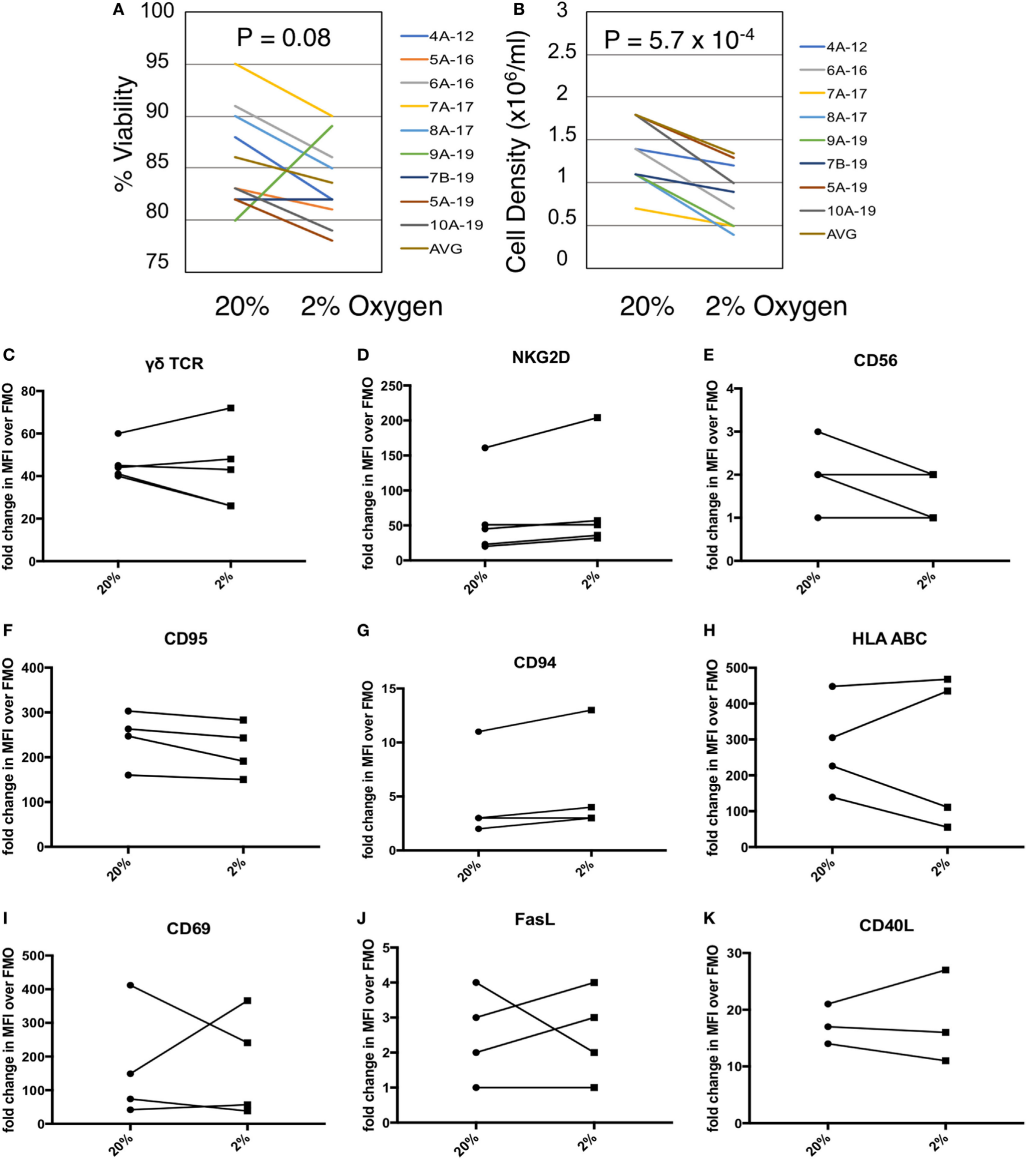
Gamma delta T cell (γδTc) viability and proliferation under hypoxia/normoxia differ, but overall surface marker expression is not significantly impacted by oxygen levels. **(A)** Viability of γδTc cultured under 20% versus 2% O_2_ for 48 h beginning on culture days 12–19, for eight cultures from seven different donors, assessed *via* Trypan Blue exclusion. Donor numbers are given with A and B indicating different cultures from the same donor; numbers after the hyphen are the culture days on which the experiment was begun. **(B)** Cell density assessment from experiment shown in **(A)**. **(C–K)** The indicated surface markers were assessed by flow cytometric analysis [**(C,D)** *n* = 5; **(E–J)** *n* = 4; **(K)** *n* = 3 different donor cultures].

**Figure 3:**
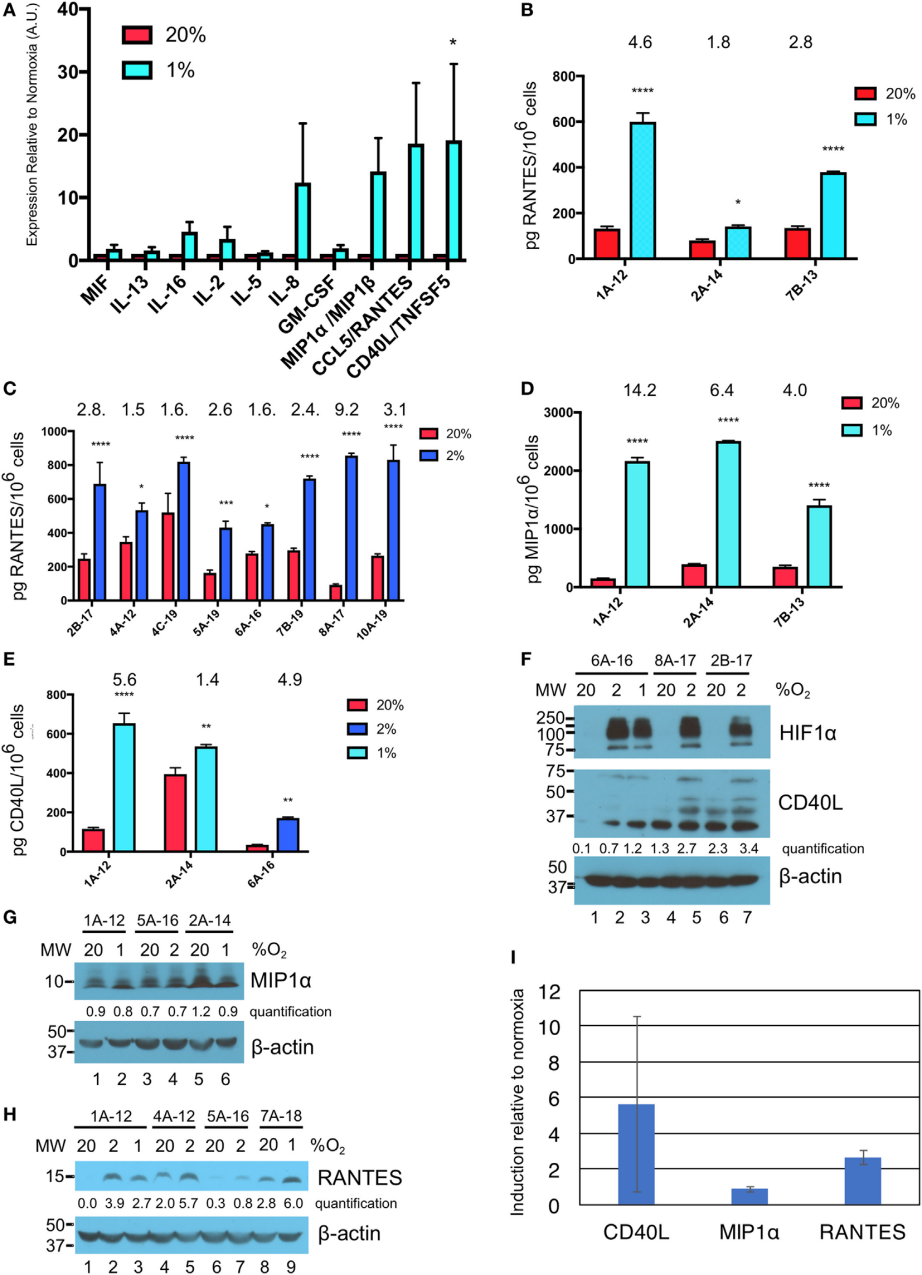
Hypoxia induces secretion of macrophage inflammatory protein 1α (MIP1α), CCL5/regulated on activation, normal T cell expressed and secreted (RANTES), and CD40L/TNFSF5 by gamma delta T cells (γδTcs). **(A)** Culture supernatants from γδTc subjected to 40 h at 20 or 1% O_2_ were analyzed by cytokine array. Cumulative results of three independent experiments for a panel of cytokines that were differentially secreted by γδTcs under hypoxia compared to normoxia are shown. Error bars are SEM; A.U. = arbitrary units; **(B)** ELISA validation of RANTES cytokine results shown in **(A)** for three independent experiments carried out at 20 and 1% O_2_ for 40 h; **(C)** RANTES ELISA for eight hypoxia experiments carried out for 48 h at 20 and 2% O_2_; **(D)** MIP1α ELISA for the same experiments shown in **(B)**; **(E)** CD40L ELISA for culture 6A-16 subject to 48 h 20 or 2% O_2_, and two of the experiments shown in **(B,D)**. Statistical analyses for **(A–E)**: two-way ANOVA, *P < 0.05, **P < 0.01, ***P < 0.001, ****P < 0.0001; **(F–H)** Western blot analysis of lysates from γδTc cultures subject to 20, 2, and/or 1% O_2_ for 48 h as indicated. γδTc culture identification is given above the blots and molecular weight (MW) markers are shown on the left; corresponding β-actin loading controls are shown in the bottom panels; relative band intensities were quantified and are indicated in arbitrary units; **(F)** three examples shown for detection of hypoxia inducible factor 1-alpha (HIF1α) (*n* = 6, 5 different donors) and CD40L (*n* = 8, 7 γδTc cultures from six donors); **(G)** MIP1α (*n* = 7, 6 γδTc cultures from five donors); **(H)** RANTES (*n* = 7); and **(I)** induction of proteins in **(F**–**H)** was determined by dividing protein band intensities from hypoxic samples by their corresponding normoxia control, and averaging these values. Error bars are SD.

**Figure 4:**
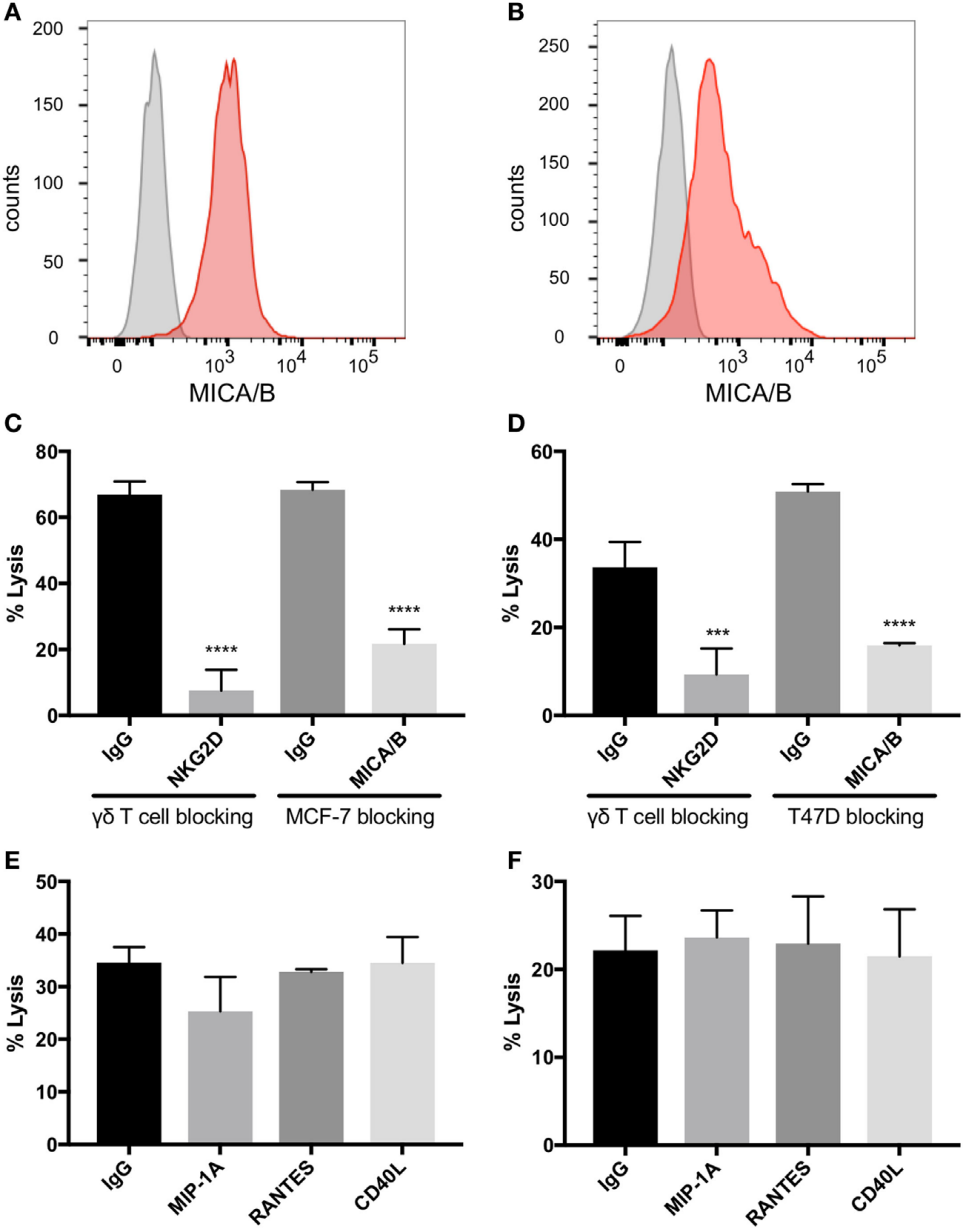
Natural killer group 2, member D (NKG2D) on gamma delta T cells (γδTcs) and MHC class I polypeptide-related sequence A (MICA)/B on breast cancer cell lines mediate γδTc cytotoxicity. Flow cytometric analysis of **(A)** MCF-7 (*n* = 4) and **(B)** T47D (*n* = 2) confirms that both cell lines express MICA/B. **(C)** Cytotoxicity assays in which NKG2D on γδTcs or MICA/B on MCF-7 cells are blocked with antibodies confirm γδTc recognition of breast cancer targets *via* this receptor/ligand interaction (*n* = 3, representative of three independent experiments with three different donor cultures). **(D)** Blocking assays as in **(C)** using T47D targets (*n* = 3). **(E)** Blocking macrophage inflammatory protein 1α (MIP1α), CCL5/regulated on activation, normal T cell expressed and secreted (RANTES), and CD40L/TNFSF5 does not decrease lysis of MCF-7 (*n* = 3 independent experiments with two different donor cultures) or **(F)** T47D (*n* = 2). Statistical analyses for **(C–F)**: one-way ANOVA, ***P < 0.001, ****P < 0.0001.

**Figure 5:**
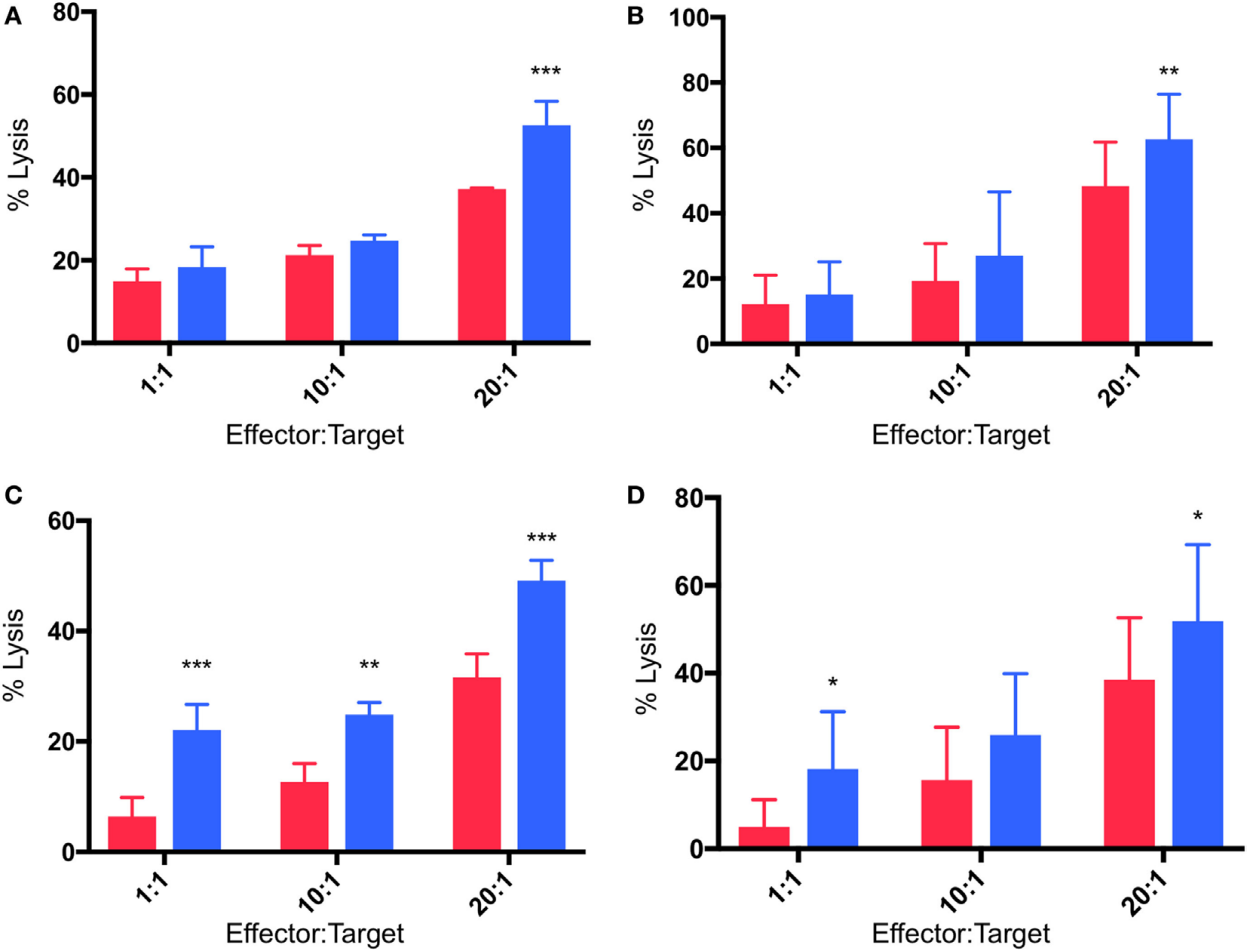
Enhanced cytotoxicity of gamma delta T cells (γδTcs) cultured in hypoxia. Cytotoxicity assays comparing γδTc cultured in 20% (red bars) or 2% O_2_ (blue bars) 48 h prior to co-culture with breast cancer target lines cultured at 20% O_2_. **(A)** A representative example of γδTc targeting MCF-7 cells; **(B)** compiled results from six independent experiments with γδTc cultures from five different donors targeting MCF-7; **(C)** a representative example with T47D targets; **(D)** compiled results from five independent experiments with γδTc cultures from four different donors targeting T47D. Two-way ANOVA, *P < 0.05, **P < 0.01, ***P < 0.001.

**Figure 6:**
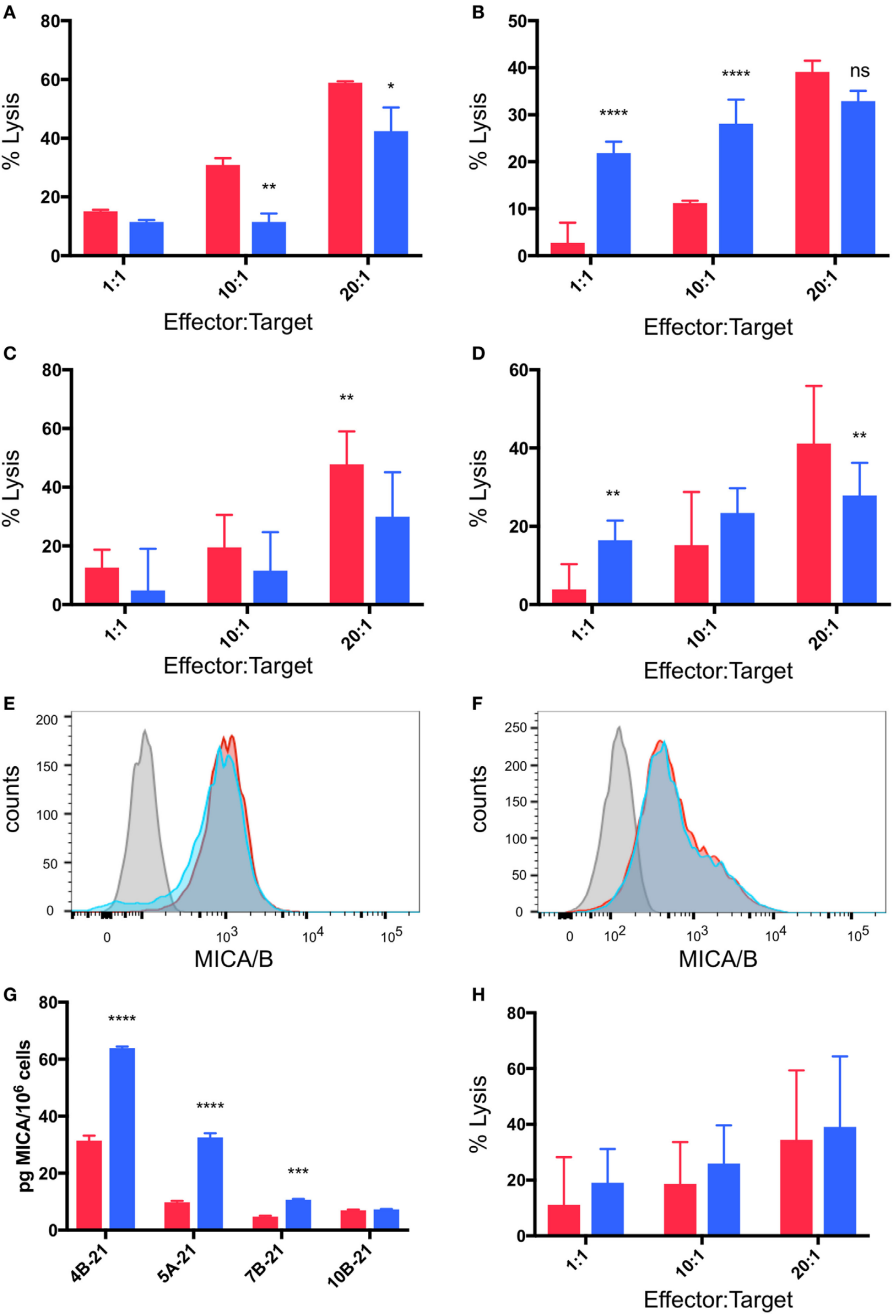
Breast cancer cell lines pre-incubated in hypoxia are resistant to gamma delta T cell (γδTc) killing. Cytotoxicity assays comparing the ability of γδTc cultured under normoxia to target breast cancer target lines cultured at 20% O_2_ (red bars) or 2% O_2_ (blue bars) for 48 h prior to co-culture under hypoxia; **(A)** a representative example in which MCF-7 cells were resistant to γδTc killing (4B-21); **(B)** an example in which MCF-7 cells cultured under 2% O_2_ were susceptible to γδTc killing (10B-21); **(C)** compiled results from five independent experiments with γδTc cultures from five different donors targeting MCF-7; **(D)** compiled results from four experiments with four different donor-derived γδTc cultures targeting T47D; **(E)** surface expression of MHC class I polypeptide-related sequence A (MICA)/B on T47D remains unchanged under hypoxia versus normoxia; **(F)** surface expression of MICA/B on MCF-7 is not differentially impacted by hypoxia versus normoxia; **(G)** MICA ELISA on concentrated supernatants of MCF-7 from experiments in **(A)**; **(H)** compiled results from five independent experiments with γδTc cultures from five different donors cultured at 20% O_2_ or 2% O_2_ targeting MCF-7 cultured under hypoxia for 48 h prior to co-culture under hypoxia. Two-way ANOVA, *P < 0.05, **P < 0.01, ***P < 0.001, ****P < 0.0001.

The following tests were used to determine significance: paired one-tailed Student’s *t*-tests [Figures 2A,B only, Microsoft Excel version 15.3 (Microsoft, Redmond, WA, USA)]; paired two-tailed Student’s *t*-tests [Figures 2C–K, Prism 7.0 for Mac OSX (GraphPad Software, San Diego, CA, USA)]; one-way ANOVA analysis (Figure 4, Prism); and Shapiro–Wilk normality tests followed by two-way ANOVA (Figure 1E, 3, 5, 6, Prism). Sidak’s pairwise multiple comparison *post hoc* tests were performed alongside ANOVA analyses. The threshold for significance was set at *P* < 0.05; asterisks indicate degrees of significance as defined in the figure legends.

## Results

### γδTc Can Be Found in Hypoxic Regions in Breast Cancer Cases

In order to determine whether γδTc are present in areas of hypoxia in breast tumors, we performed immunohistochemistry to detect the hypoxia marker CAIX and γδTc using single stains of serial sections from a panel of 17 breast tumors (Table 1). Examples from one case (case 14) are shown (Figures 1A–D), including images of a CAIX-positive region (Figure 1A), an area with no appreciable CAIX positivity (Figure 1B), and increased magnification of γδTc found in the same region depicted in Figure 1A (Figure 1C) and Figure 1B (Figure 1D). Of these 17 cases, 47% (8/17) stained positively for CAIX. In CAIX-negative cases, there was little γδTc infiltration; however, when γδTc were quantified in CAIX-positive versus CAIX-negative areas of breast tumors, γδTc frequency was greater in hypoxic regions, significantly so in three cases in particular (Figure 1E, cases 13, 14, and 17, *P* < 0.0001). Images for cases 13 and 17 are in Figure S1 in Supplementary Material. In our cohort, 71% (12/17) of tumors were estrogen receptor positive (ER+); most ER+ cases were CAIX-negative (Figure 1E, ER status indicated below case numbers).

### Exposure to Hypoxia Reduces γδTc Density

Given the co-localization of γδTc and CAIX in breast tumors, we measured the effects of hypoxia on γδTc viability and density *in vitro*. We cultured γδTc for 12–19 days, then subjected them to 48 h in hypoxic (2% O_2_) or normoxic (20% O_2_) conditions. We found that exposure to hypoxia had variable effects on γδTc viability (Figure 2A, *P* = 0.08), and significantly decreased cell density (Figure 2B, *P* = 5.7 × 10^−4^). Immunophenotyping was performed using flow cytometric analyses of activation markers including γδTCR, NKG2D, CD56, CD69, CD95, CD40L, and HLA ABC as well as the inhibitory markers FasL and CD94. γδTc were stained with live/dead ZA prior to surface marker staining. Median fluorescence intensity values (MFIs) of hypoxia and normoxia samples were divided by the MFI of FMO controls to obtain fold-change values. Surface markers on γδTc cultures subjected to 48 h 20 or 2% O_2_ were not significantly different (Figures 2C–K).

### MIP1α, RANTES, and CD40L Are Secreted by γδTc in Hypoxia

Culture supernatants from three different donor γδTc cultures subject to 40 h of normoxia or hypoxia were analyzed by cytokine array. While IL-8 appears elevated in the cumulative results graph depicted here (Figure 3A), this cytokine was only greatly increased under hypoxia in one of three experiments (Figure S2B in Supplementary Material, *P* < 0.0001), was moderately increased in one experiment (Figure S2A in Supplementary Material, *P* < 0.05), and not significantly elevated in the third experiment (Figure S2C in Supplementary Material). Due to significant variation among donor cultures, cumulative results reveal significantly increased secretion of only CD40 ligand (CD40L or CD154) under hypoxia compared to normoxia (Figure 3A, *P* = 0.0472). However, in all three individual cytokine arrays, significantly increased secretion of MIP1α [or CCL3 = chemokine (C–C motif) ligand 3], RANTES (or CCL5), and CD40L under hypoxia compared to normoxia was observed (Figures S2A–C in Supplementary Material). Note that equal cell numbers were plated, and relative values at 2 and 1% O_2_ were normalized to normoxia without taking harvested cell numbers into account. Considering the decrease in γδTc densities observed under hypoxia, this suggests an even greater effect would be observed if comparing the output of equal cell numbers.

ELISA validation for expression of RANTES, MIP1α, and CD40L was performed with culture supernatants from three different γδTc cultures (Figures 3B–E, hypoxia = 1 or 2% O_2_ as indicated). For RANTES expression, an additional eight experiments were assayed, for secretion over 48 h at 20 or 2% O_2_ (Figure 3C). In this case, and in contrast to the cytokine array data, ELISA values were normalized to cell numbers. Significantly increased secretion of these cytokines by γδTc was observed when cells were cultured in hypoxia compared to normoxia (asterisks indicate significance). A wide range of average secreted RANTES levels was observed, ranging from 93 to 521 pg/million γδTc in normoxia to 431 to 856 pg/million γδTc under hypoxia; the average ratio hypoxia:normoxia is indicated above the bars (Figures 3B–E). Likewise, secreted MIP1α and CD40L levels were quantified for three independent experiments using ELISA (Figures 3D,E). MIP1α levels ranged from 152 to 394 pg/million γδTc in normoxia to 1,406 to 2,509 pg/million γδTc under hypoxia, with fold changes from 4.0 to 14.2 (Figure 3D). Similarly, CD40L secretion by γδTc increased significantly when cultured in low O_2_, with 2% O_2_ in one experiment yielding an average of 171 pg CD40L/million γδTc in hypoxia, a 4.9-fold increase over just 35 pg CD40L/million γδTc in normoxia (Figure 3E). Two experiments conducted with 1% O_2_ yielded a wide range of CD40L secretion by γδTc in both conditions (Figure 3E, 120–395 and 536–653 pg CD40L/million γδTc in normoxia and hypoxia, respectively).

Western blotting was done to verify induction of HIF1α in γδTc under hypoxia, and also to investigate whether intracellular levels of CD40L, MIP1α, and RANTES reflected those of secreted proteins (Figures 3F,G). HIF1α was clearly induced in γδTc at 2 and 1% O_2_ in all cases; three examples from six independent experiments with five donor cultures are shown (Figure 3F, top panel, compare lane 1 versus 2 and 3, 4 versus 5, and 6 versus 7). CD40L appears visibly increased in hypoxia samples for γδTc culture 6A-16 (Figure 3F, middle panel, compare lane 1 versus 2 and 3), and quantification suggests this is also the case for the other two donor cultures shown (lane 4 versus 5 and lane 6 versus 7). Note that several forms of CD40L are evident here, which were included in the quantification of bands. Of eight experiments with seven γδTc cultures from six donors, intracellular CD40L was clearly visibly increased in three (38%). HIF1α and CD40L blots originated from the same gel, which was transferred and then cut at 75 kDa; thus, the β-actin loading control serves for both (Figure 3F, lower panel). MIP1α levels were not consistently higher in γδTc subject to hypoxia versus normoxia (Figure 3G, representative of seven experiments with six γδTc cultures from five donors), as demonstrated by very similar quantification values within each experiment. By contrast, RANTES was typically induced by hypoxia, with higher protein levels evident in cellular lysates from γδTc cultured in 1 or 2% O_2_ compared to normoxia (Figure 3H, compare lane 1 versus 2 and 3, 4 versus 5, and 8 versus 9; *n* = seven independent experiments, seven donors, induction clear in six, unclear in one). Longer exposure of this blot also revealed RANTES induction in lane 7 versus 6 (Figure S3 in Supplementary Material). Full scans of Western blots can be found in Figure S4 in Supplementary Material. The average induction of CD40L, MIP1α, and RANTES in γδTc under hypoxia relative to normoxia was calculated using Western blot band intensity values, and confirmed elevated levels of intracellular CD40L and RANTES, but not MIP1α, under hypoxia (Figure 3I).

### NKG2D Expressed on γδTc and MICA/B on Breast Cancer Targets Are Critical for γδTc Killing

MCF-7 and T47D are estrogen receptor (ER) positive luminal A breast carcinoma cell lines (35). Both of these cell lines express MICA/B on the surface as identified by flow cytometric analysis (Figures 4A,B). Blocking NKG2D on γδTc significantly decreased lysis of MCF-7 (Figure 4C, one-way ANOVA versus IgG control, *P* < 0.0001, representative of four independent experiments, *n* = 4) and T47D (Figure 4D, *P* = 0.0002, *n* = 5). Likewise, blocking the NKG2D ligand MICA/B on targets prevented MCF-7 and T47D cell lysis (Figures 4C,D, both *P* < 0.0001, *n* = 2 and 3, respectively). By contrast, no decrease in cell lysis of either line was observed when γδTc were pre-incubated with antibodies against MIP1α, RANTES, or CD40L (Figures 4E,F, *n* = 3 and 2, respectively). Since antibodies were not washed away prior to co-incubation with targets, blocking should have been effective against both membrane-bound and soluble proteins. Thus, it appears that MIP1α, RANTES, and CD40L are not directly involved in γδTc cytotoxicity against MCF-7 or T47D.

### γδTc Cytotoxicity Against MCF-7 and T47D Targets Is Enhanced in Hypoxia

Cytotoxicity experiments were performed in which γδTc effectors and breast cancer cell lines were pre-incubated for 48 h under normoxia or hypoxia (2% O_2_) and then co-cultured at 1:1, 10:1, and 20:1 E:T ratios in parallel under normoxia or hypoxia, as per target pre-incubation conditions, for 4 h. Pre-incubation in hypoxia enhanced γδTc cytotoxicity against MCF-7 targets cultured in normoxia (Figures 5A,B). In a representative example, significantly increased MCF-7 cell lysis was observed at 20:1 (Figure 5A, *P* = 0.0005); when data from all six experiments performed with day 21 γδTc from five different donors (six different cultures) were compiled and subject to statistical analysis, this result was confirmed (Figure 5B, *P* = 0.007). Likewise, γδTc cultured in hypoxia were better able to kill T47D cultured in normoxia (Figures 5C–D). In an example representative of five experiments with day 21 γδTc from four different donors, target cell lysis was significantly increased at all E:T ratios tested (Figure 5C, *P* < 0.01); analysis of compiled results from all five experiments revealed significantly increased lysis of targets by hypoxia-treated γδTc at 1:1 and 20:1 E:T (Figure 5D, *P* < 0.05).

### Breast Cancer Targets in Hypoxia Are Resistant to γδTc Killing due to MICA Shedding

As outlined above, cytotoxicity experiments were performed in which breast cancer cell lines were pre-incubated for 48 h under normoxia or hypoxia (2% O_2_) and then co-cultured with γδTc at 1:1, 10:1, and 20:1 E:T in parallel under normoxia or hypoxia for 4 h. In most cases (4/6, 67%), pre-incubation in hypoxia induced MCF-7 resistance to γδTc cytotoxicity (Figures 6A–C). In a representative example from an experiment performed with γδTc culture 4B-21, significantly decreased MCF-7 cell lysis was observed at 10:1 (Figure 6A, *P* = 0.0054) and 20:1 (Figure 6A, *P* = 0.0119). By contrast, in two experiments with two different γδTc cultures from the same donor, no resistance was observed; one example is shown in which MCF-7 cultured under hypoxia appeared to be more susceptible to γδTc killing (Figure 6B, *P* < 0.0001 at 1:1 and 10:1). When data from five experiments performed with day 21 γδTc from five different donors were compiled and subject to statistical analysis, the overall effect of hypoxia inducing MCF-7 resistance was confirmed (Figure 6C, *P* = 0.0011). Likewise, T47D cultured in hypoxia were more resistant to γδTc killing at 20:1 than those cultured in normoxia (Figure 6D, *P* = 0.0043), although the 1:1 result is opposite (*P* = 0.0076); these compiled results were from four experiments conducted with four different γδTc donor cultures. Flow cytometric analysis of MICA/B surface expression on breast cancer lines subjected to 48 h normoxia or hypoxia revealed no significant change in MFI; representative examples are shown for MCF-7 (Figure 6E, *n* = 4) and T47D (Figure 6F, *n* = 2). Of note, Accutase was used for dissociation of these adherent cell lines, out of concern for potential trypsin sensitivity of surface MICA/B that might have confounded our results. Supernatants from MCF-7 and T47D subject to 48 h 20 or 2% O_2_ were subject to MICA ELISA (Figure 6G). MICA could not be detected in supernatants directly, thus samples were concentrated and MICA ELISA was repeated. MICA in T47D remained below the detection limit; however, after normalization to cell numbers, a significant increase in secreted MICA by MCF-7 cells under hypoxia was observed in 3/4 experiments (Figure 6G, ***P = 0.0005, ****P < 0.0001). These results match those observed in cytotoxicity experiments, with ELISA from MCF-7 targets used in cytotoxicity assays with 4B-21 showing increased MICA secretion under hypoxia that fits with the observed resistance to γδTc cytotoxicity in Figure 6A. Likewise, no difference in MICA secretion was observed in MCF-7 targets under 20 or 2% O_2_ subject to cytotoxicity assays with γδTc culture 10B-21, which also showed no MCF-7 resistance to γδTc killing in Figure 6B. Thus, resistance to γδTc killing appears to be correlated with MICA secretion by breast cancer targets. Despite enhanced cytotoxicity of γδTc cultured under 2% compared to 20% O_2_ against targets cultured under normoxia (Figure 5), they are unable to overcome resistance exhibited by MCF-7 under 2% O_2_, as revealed by analysis of five compiled experiments comparing γδTc cultured under 20 or 2% O_2_ against MCF-7 cells cultured in hypoxia (Figure 6H).

## Discussion

Gamma delta T cells are being developed as immunotherapeutic agents for a variety of cancer indications and clinical trials (Phase I/II) thus far have shown excellent safety profiles (36). Yet, they are known to embody remarkable functional plasticity, dependent on the environment in which they find themselves (24, 37–39). Thus, it is important to explore the function of γδTc infiltrating solid tumors, some of which may be hypoxic. In our small cohort of 17 breast cancer cases, 47% of tumors contained areas of CAIX positivity indicating hypoxia (Figure 1). The CAIX-negative cases were 89% ER+ (Figure 1E, cases 1–9, case 7 was of unknown ER status); of ER+ cases, 76% were CAIX negative. This confirms reports showing up to 80% CAIX negativity in studies assessing ER+ breast tumors; in these cases, CAIX negativity correlated with low histological grade (40). While our cohort was admittedly small, the very low levels of γδTc infiltrates in CAIX-negative tumors, correlated with low histological grade, confirm results showing that levels of γδTc infiltration correlate positively with higher histological grades (41). Unfortunately, our cohort size was too limited to determine whether γδTc infiltration correlated with patient outcome. We did, however, find γδTc in areas of hypoxia in some tumors. While we did not have the power in our study, or *in vivo* functional data, to claim that γδTc are preferentially attracted to hypoxic regions, our results at least provide an indication that γδTc can be found in hypoxic areas of tumors, and that studying their function under low O_2_ is worthwhile. As CAIX is associated more so with triple negative breast cancers (TNBC) (18, 42), future studies of γδTc and hypoxia should focus on a larger cohort of TNBC patients. Indeed, the groundwork for such studies has been laid by Hidalgo and colleagues, who recently reported on the pattern of distribution of γδTc in TNBC (43).

It was unsurprising that γδTc cell density decreased under hypoxia (Figure 2), as terminally differentiated γδTc stop proliferating to become cytotoxic (44), and hypoxia enhanced γδTc cytotoxicity (Figure 5). Delayed cell-cycle progression was also noted in a study on PBMC in hypoxia (45). To our knowledge, the only study of γδTc in the context of hypoxia showed that circulating γδTc in patients with obstructive sleep apnea had elevated intracellular tumor necrosis factor alpha (TNFα) and IL-8 levels, increased TNFα and L-selectin-mediated adhesion properties, and enhanced cytotoxicity against endothelial cells compared to those isolated from healthy donors (46). While that study compared freshly isolated blood-derived γδTc from patients and healthy donors, we used healthy donor-derived *in vitro* expanded γδTc for our experiments, which potentially accounts for different results. TNFα secretion was not impacted by hypoxia in our study, as no differential effects were detected by cytokine array (data not shown). While we did observe strongly elevated hypoxia-induced IL-8 in the supernatant of one of the three γδTc cultures subject to cytokine array analysis (Figure S2 in Supplementary Material), this was not the case for the other two cultures.

More significant were cytokine array data pointing to increased secretion of RANTES, MIP1α, and CD40L by γδTc under low O_2_ compared to normoxia that were confirmed by subsequent ELISAs (Figures S2A–C in Supplementary Material; Figures 3B–E). Intracellular protein levels induced by hypoxia matched ELISA results only in the case of RANTES (Figure 3H); the same could not be said for CD40L and MIP1α, where hypoxia treatment did not appear to increase intracellular levels (Figures 3F,G), and surface expression of CD40L was variable (Figure 2K). Since blocking these proteins appeared to have no impact on γδTc cytotoxicity against breast cancer target lines (Figures 4E,F), they must have an indirect function related to enhanced cytotoxicity of γδTc under hypoxia.

Human memory Vγ2Vδ2 cells were reported to store cytoplasmic RANTES that was secreted rapidly in response to TCR signaling, but little MIP1α protein was found in these cells (47). RANTES is a chemokine employed to recruit antigen presenting cells, such as dendritic cells (48, 49), and thus speaks to the anti-tumor function of γδTc in hypoxia, though breast tumors may use this to their own advantage to promote malignancy (50). RANTES and MIP1α expression were also reported to aid Vδ1 cell suppression of HIV replication (51). CD40 ligation is thought to enhance the immunogenicity of tumors (52), thus γδTc may secrete CD40L in order to better “see” tumor targets. CD40L may also inhibit growth of CD40-expressing tumors directly (52–55). Further investigation will be required to determine the functions served by these cytokines with respect to γδTc targeting solid tumors.

A study of the Vγ9Vδ2 γδTc subset in the context of breast cancer suggested that surface levels of MICA/B on breast cancer target cell lines were associated with γδTc cytotoxicity against these lines; however, direct blocking assays were not carried out (16). Both MCF-7 and T47D cells expressed surface MICA/B, in contrast to an earlier report suggesting a lack of MICA/B expression on MCF-7 (56). If trypsin was used to dissociate MCF-7 in that study, it might explain their inability to detect MICA/B; to avoid this issue, we used Accutase to dissociate our adherent cell lines, as detachment of cells is gentler and protects most surface epitopes. We have confirmed the involvement of NKG2D on γδTc and MICA/B on MCF-7 and T47D in cytotoxicity of γδTc against breast tumor targets (Figure 4), although differences in the ability of γδTc to kill targets pre-incubated in hypoxia or normoxia do not appear to be related to surface levels of MICA (Figure 6).

One mechanism of hypoxia-mediated tumor evasion is MICA shedding (57). MICA downregulation related to shedding under hypoxia, as well as downregulated expression of NKG2D on PBMCs incubated with culture supernatants of prostate cancer cells exposed to hypoxia—abrogated upon incubation with MICA blocking antibodies—has been reported (58). MICA shedding is not a universal evasion mechanism employed by all cancer cells, however, as glioblastoma cell lines did not shed MICA, although this study was only carried out under normoxia (59). While we assume that soluble MICA may bind NKG2D and block or downregulate this receptor to prevent γδTc recognition of breast cancer targets, a recent report suggests that, in mice, soluble NKG2D might activate NK cells and aid in tumor eradication, but this anti-tumor effect has yet to be shown in humans or with γδTc (60). By contrast, soluble MIC was shown to decrease γδTc cytotoxicity in pancreatic cancer (61) and has been implicated in evasion of human ovarian cancer cells from γδTc recognition (21). Thus, we were surprised that surface expression of MICA/B on MCF-7 and T47D breast cancer lines appeared unaffected by 48 h under hypoxia (Figure 6). However, MICA secretion did not correlate with MICA surface levels, as soluble MICA increased in the supernatants of MCF-7 cells cultured under hypoxia, while surface MICA levels remained unchanged (Figure 6). Thus, it appears that we would need to neutralize soluble MICA to improve γδTc cytotoxicity, since target surface expression did not appear to be affected by hypoxia. That said, we did not directly assess MICA expression during co-culture with γδTc, and it is possible that MICA was downregulated in the presence of γδTc, although the correlation between resistance to γδTc killing and soluble MICA levels in culture supernatants under hypoxia speaks against this (Figure 6). One way to overcome MICA shedding may be to increase nitric oxide signaling (58), although its impact on γδTc would have to be assessed.

Although the γδTc tumor infiltrating lymphocytes (TIL) signature was deemed the most positive prognosticator across a range of cancers, including breast cancer (62), some reports suggest that γδTc may take on a regulatory phenotype within the breast TME (41, 56, 63, 64). In one study, γδTc TIL isolated from a breast tumor were expanded in high levels of IL-2 for several weeks prior to immunosuppression assays and proved to inhibit dendritic cell maturation and CD8+ T cell cytotoxicity (56); however, given the known functional plasticity of γδTc, such assays conducted on *ex vivo* expanded cells removed from the TME cannot inform the function of γδTc *in situ*. A positive correlation was observed between γδTc infiltration and breast cancer stage, leading the authors to suggest that γδTc may contribute to disease pathology; however, causality was not established (41). Although our cohort size was much smaller, we too observed a positive correlation between CAIX expression, indicating hypoxia—typically an indicator of cancer progression—and γδTc infiltration (Figure 1). This could just as easily indicate the greater need for γδTc attempting to eradicate disease. Our hypoxia experiments reveal enhanced cytotoxicity of γδTc exposed to 48 h of low O_2_, suggesting that γδTc are indeed able to kill in this environment (Figure 5). Soluble MICA appears to inhibit γδTc cytotoxicity against breast tumor targets in hypoxia and, despite their increased killing capacity under low O_2_, γδTc are unable to overcome resistance exhibited by MCF-7 under 2% O_2_ (Figure 6), a condition under which γδTc must operate within at least some parts of a tumor. Further studies will be required to definitively identify γδTc function in breast tumors *in situ*.

## Ethics Statement

This study was carried out in accordance with the recommendations of the Research Ethics Guidelines, Health Research Ethics Board of Alberta—Cancer Committee with written informed consent from all subjects. All subjects gave written informed consent in accordance with the Declaration of Helsinki. The protocol was approved by the Health Research Ethics Board of Alberta—Cancer Committee.

## Author Contributions

GS and L-MP contributed to research design. GS and ID conducted experiments; data analysis was carried out by GS, ID, and RL. GS wrote the manuscript; all authors provided feedback and approved the final version.

## Conflict of Interest Statement

The authors declare that the research was conducted in the absence of any commercial or financial relationships that could be construed as a potential conflict of interest.

## Abbreviations

BP, band pass; CAIX, carbonic anhydrase IX; CalAM, Calcein AM; CD40L, CD40 ligand (or CD154); E:T, effector:target ratio; ER, estrogen receptor; FBS, fetal bovine serum; FMO, fluorescence minus one; γδTcs, gamma delta T cells; HIF1α, hypoxia inducible factor 1-alpha; HRP, horseradish peroxidase; IL, interleukin; LP, long pass; MICA, MHC class I polypeptide-related sequence A; MIP1α, macrophage inflammatory protein 1α [or CCL3 = chemokine (C–C motif) ligand 3]; MFI, median fluorescence intensity; NKG2D, natural killer group 2, member D; O_2_, oxygen; PBMCs, peripheral blood mononuclear cells; PBS, phosphate buffered saline; PIC, protease and phosphatase inhibitor cocktail; PR, progesterone receptor; RANTES, regulated on activation, normal T cell expressed and secreted (or CCL5); TBST, tris-buffered saline plus 0.05% Tween-20; TCR, T cell antigen receptor; TIL, tumor infiltrating lymphocytes; TME, tumor microenvironment; TNFα, tumor necrosis factor alpha; ZA, Zombie Aqua fixable viability dye.
