# Supplementary figures and images for: Functional Plasticity of Gamma Delta T Cells and Breast Tumor Targets in Hypoxia
Source: Front Immunol. 2018 Jun 15;9:1367. doi: 10.3389/fimmu.2018.01367 (PMC6013583; doi:10.3389/fimmu.2018.01367)

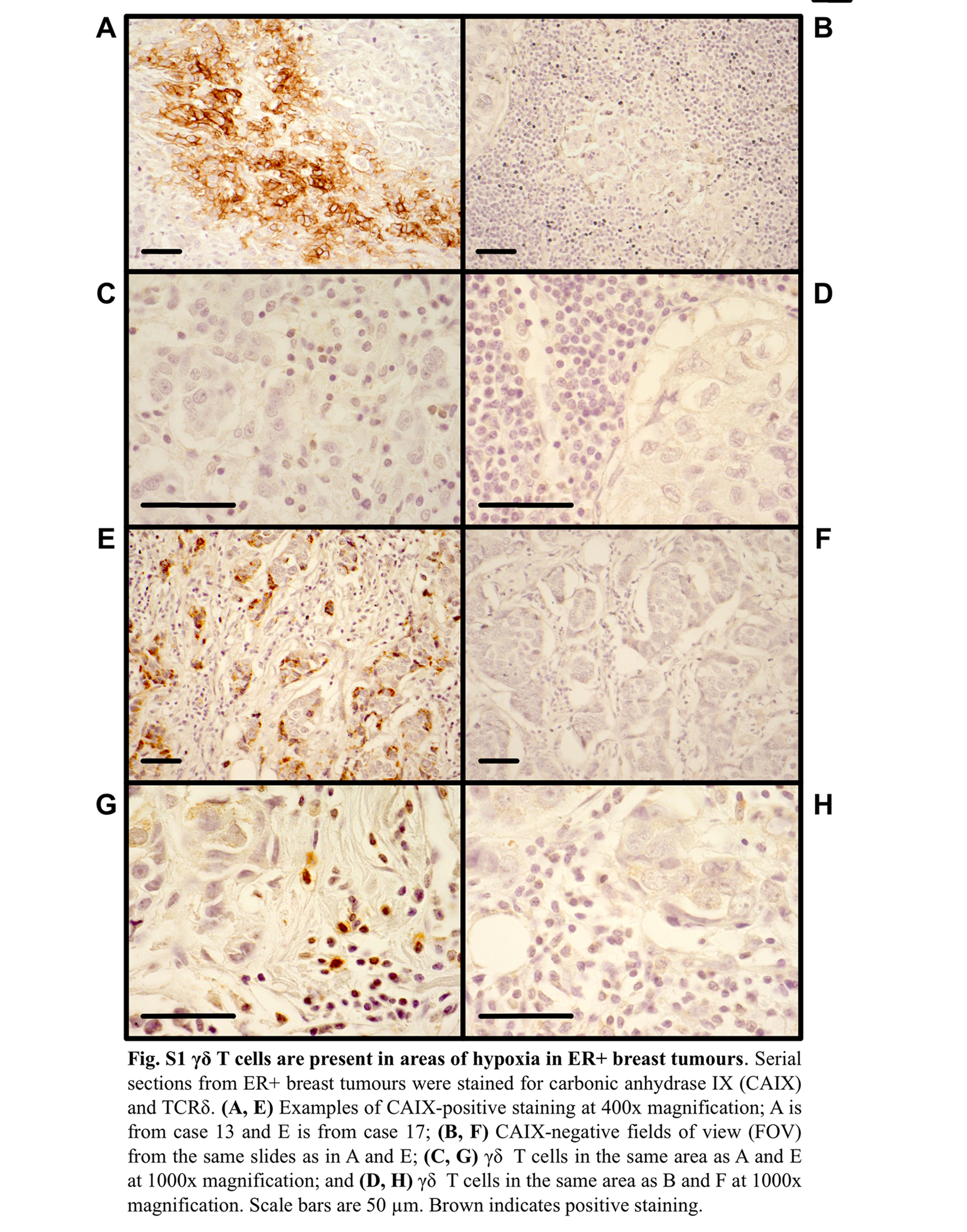

Supplement: Supplementary file 1 [file Image_1.tif]

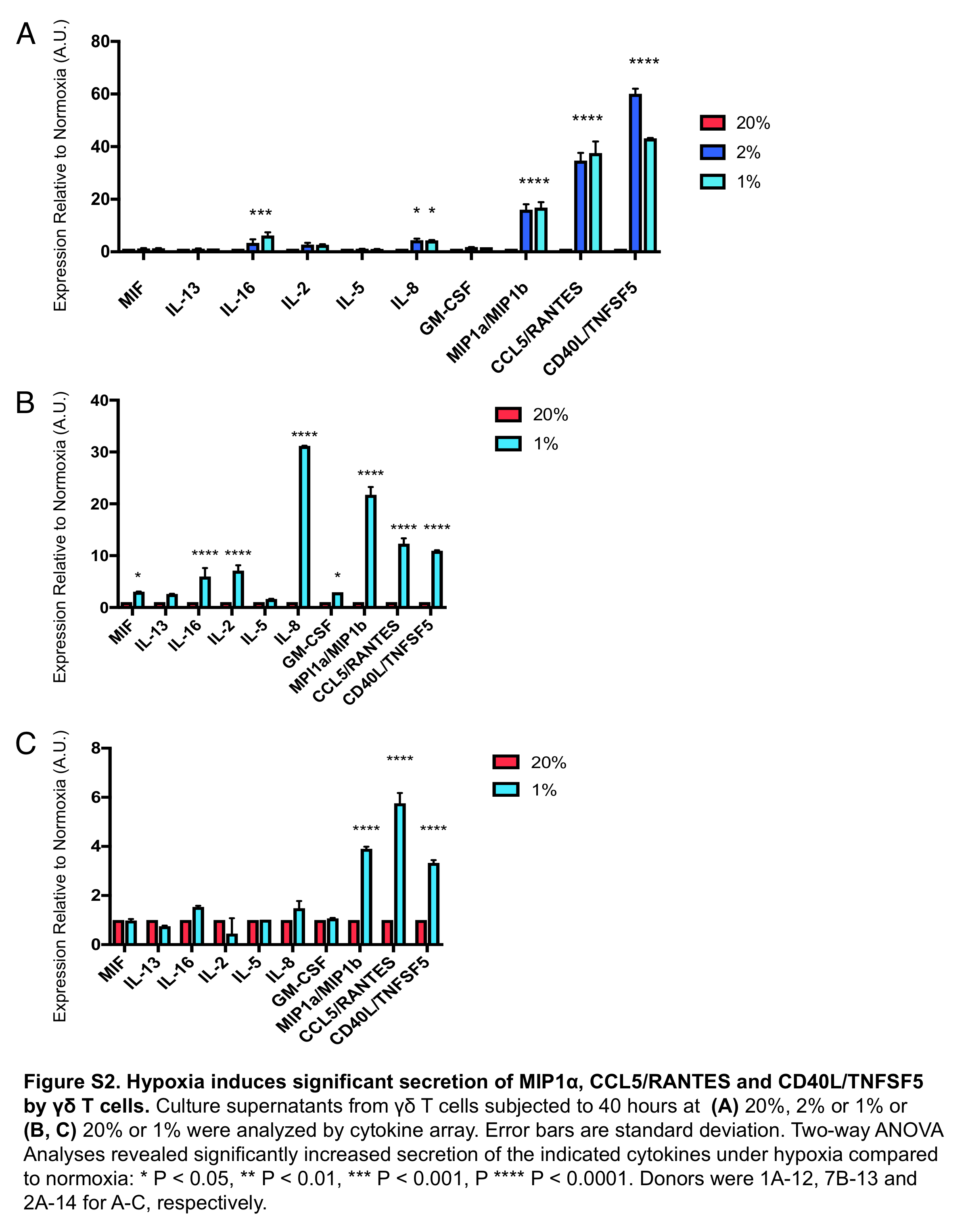

Supplement: Supplementary file 2 [file Image_2.tiff]

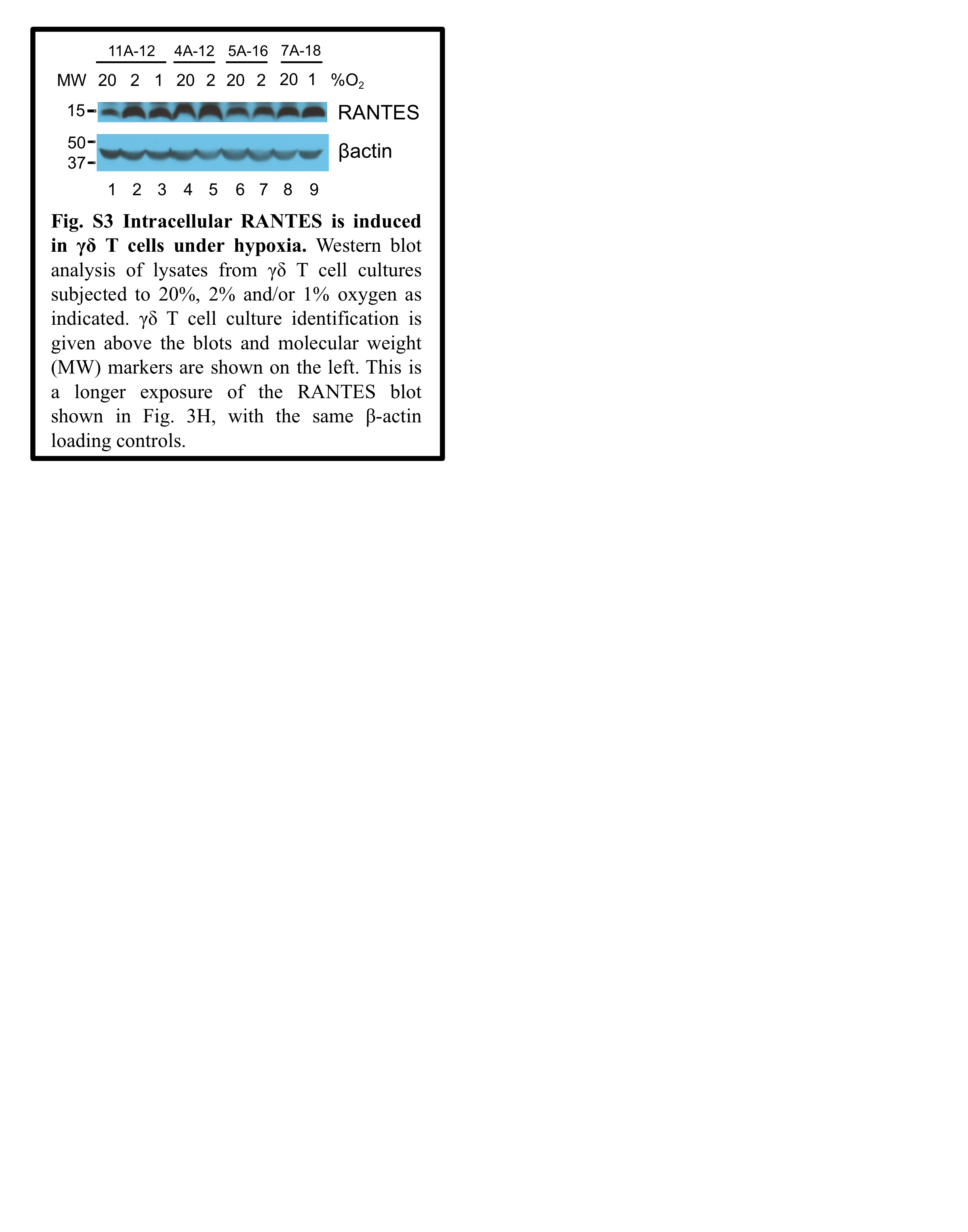

Supplement: Supplementary file 3 [file Image_3.tiff]

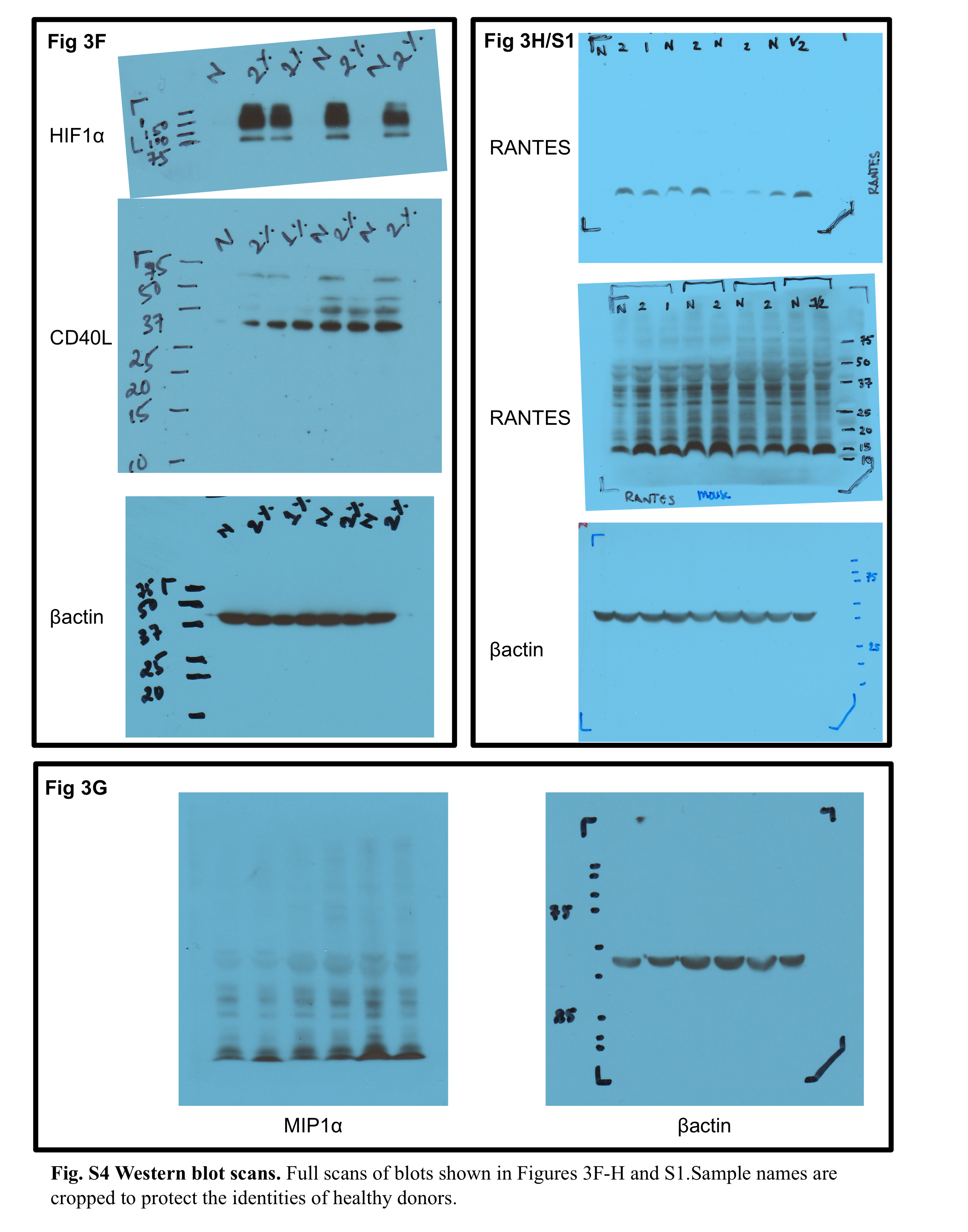

Supplement: Supplementary file 4 [file Image_4.tiff]
