# Supplementary material for: Functional Plasticity of Gamma Delta T Cells and Breast Tumor Targets in Hypoxia
Source: Front Immunol. 2018 Jun 15;9:1367. doi: 10.3389/fimmu.2018.01367 (PMC6013583; doi:10.3389/fimmu.2018.01367)
Supplement: Supplementary file 5 [file Table_1.docx]

| ID | Day | % Vδ1 | % Vδ2 | % γδTCR+Vδ1-Vδ2- | % Purity | Figure(s) |
| --- | --- | --- | --- | --- | --- | --- |
| 1A | 21 | 23.2 | 69.3 | 5.5 | 98.0 | 3A,B,D,E,G,H |
| 2A | 17 | 11.6 | 74.8 | 1.8 | 88.2 | 3B,D,E,G |
| 2B | 19 | 5.4 | 91.0 | 0.9 | 97.3 | 3C,F |
| 4A | 12 | 47.0 | 22.4 | 11.0 | 80.4 | 2C-F; 3C,H |
| 4B | 21 | 36.3 | 42.1 | 6.0 | 84.4 | 5B,D; 6C,D,G,H |
| 4C | 21 | 20.1 | 58.0 | 5.1 | 83.2 | 3C; 4E,F |
| 5A | 21 | 11.4 | 78.8 | 4.2 | 94.4 | 2C-F; 3C,G,H; 4C,D; 5B,D; 6C,D,G,H |
| 6A | 21 | 12.1 | 79.3 | 3.4 | 94.8 | 2C-F; 3C,E,F |
| 7A | 21 | 1.1 | 86.7 | 3.6 | 91.4 | 2C-F; 3H |
| 7B | 19 | 2.3 | 88.9 | 2.7 | 93.9 | 2C,D; 3B-D; 5B,D; 6C,G,H |
| 8A | 21 | 42.0 | 46.2 | 9.5 | 97.7 | 2C-F; 3C,F |
| 9A | 21 | 6.6 | 85.0 | 2.7 | 94.3 | 2C,D; 5B; 6C,H |
| 10A | 21 | 7.5 | 73.3 | 2.5 | 83.3 | 2C,D; 3C; 5A,B,D; 6C,D |
| 10B | 21 | 19.9 | 64.0 | 3.7 | 87.6 | 5B-D; 6B,D,G,H |

**Table S1** Edmonton donor γδ T cell culture subset percentages and purities. Cells were harvested on the indicated day, stained with Zombie Aqua fixable viability dye followed by antibodies recognizing pan γδ TCR, Vδ1 TCR and Vδ2 TCR and acquired by flow cytometry. The sum of %Vδ1, %Vδ2 and %γδTCR+Vδ1-Vδ2- is the purity. Figures in which results from these cultures appear are listed.
